# Pulpless Teeth; Abscess; Treatment, Especially Surgical Treatment

**Published:** 1892-04

**Authors:** J. M. Porter

**Affiliations:** New York


### PULPLESS TEETH; ABSCESS; TREATMENT, ESPECIALLY SURGICAL TREATMENT.[1]

BY J. M. PORTER, D.D.S., NEW YORK.

EVERY man beginning dental practice doubtless has his vision of the methods by which he can most quickly and surely relieve the pathological conditions coming under his professional care.

This vision may be the impressions made by a preceptor, or the result of clinical teaching. In either event his individuality will in time so influence his methods, that two men having the same preceptor and graduating in the same class will in a very few years pursue radically different procedures in attaining a given result. That men of large experience should differ widely as to the best methods by which a pulpless tooth can most surely be placed in a condition to be safely filled is not surprising. Thus, one endowed

---

[1] Read before the New York Odontological Society, January 19, 1892.

with conservative tendencies will unconsciously employ conserva-tive means, while the man with more radical proclivities will adopt very different methods to attain the same result, and the process adopted by each will very generally bear the impress of his individuality.

In my early experience I pursued the more conservative plan, chiefly because my clinical teaching had been in that direction, but time and experience taught me there was a more excellent mode. I then thought it necessary to disinfect the pulp-canals and tubuli of a pulpless tooth for several days, or perhaps weeks, without regard to the physical conditions surrounding the case. For instance, no distinction was made between a case where the pulp had been dead for several months and there had been pericementitis, and the case in which the pulp had been dead but a short time without symptoms of inflammation. I did not take into consideration the age of the patient, which is a factor of much importance, and one not regarded as it should be in making a diagnosis. In those days I used creosote or carbolic acid as my usual treatment, but for the past five years I have used neither in pulpless teeth, with or without abscess. In the first class I will include those in which the pulps have recently died or been destroyed. In this some difficulty will be experienced in removing satisfactorily all of the pulp. Formerly, after removing the pulp, this class of cases was treated with carbolic acid or creosote, and when the patient returned a substance was found in the smaller portions of the pulp-canal, very sensitive to the touch of a broach and incapable of absorbing any remedy which I could use for the purpose of relieving this condition.

I now remove, if possible, all the pulp at one sitting, and immediately fill the pulp-canals. By the use of cocaine this result is accomplished without producing much discomfort or pain.

In the next class I will include those teeth with dead pulps which have never manifested any symptoms of pericemental inflammation. In such cases, especially if the patient has passed the age of forty years, I do not hesitate to fill at the time of second treatment, and in a few a filling is made at the first sitting. For instance, a patient of the last class mentioned called for treatment within a month. I found a large anterior approximal decay in the first right superior molar. The nerve had evidently been dead several months. There was no soreness on percussion, and after adjusting the rubber-dam and thoroughly cleansing the cavity, I found no defined pulp-canals. I thoroughly dried with hot air, then washed the entire cavity with alcohol, repeating this treatment a number of times at this sitting, and filled immediately with gold, without having any subsequent unfavorable symptoms. This belonged to the class in which I regard immediate root-filling as being clearly indicated.

The next class will include pulpless teeth with soreness on percussion. Here would be an evidence of pericementitis, slight or serious, depending upon the degree of tenderness manifested, the significance of which experience alone will indicate. I am advised by a well-known dentist in this city, that in treating this class he never attempts to remove all the dead pulp at one sitting, but permits a portion to remain, treating with iodoform and sealing up the cavity for subsequent operation. I have always had the impression that disinfecting the tubuli was the chief object in the management of teeth in this condition, and certainly the chief motive where the pulp had evidently been dead for several months. How this process of disinfecting the tubuli can be best accomplished by leaving in the pulp-canal a portion of the dead pulp, through which the iodoform would have to act upon the tubuli, is a surgical proposition which I am not able to comprehend. I believe in getting direct contact with the tubuli and then using hot air to evaporate all the moisture possible, so that the process of disinfection can be hastened. The pulp-canals are then washed several times with alcohol, alternating the alcohol with the use of hot air, which method I believe to most quickly produce the conditions desired, so that the operation of filling can be accomplished without causing subsequent irritation. In the three classes of cases to which I referred I seriously object to leaving the pulp-canals unfilled any longer than is absolutely necessary, because this condition is radically unnatural, and it is a well-known fact in surgery that nature makes every effort to remove all unnatural or pathological conditions. The forming of a scab over a wound is a good illustration of the efforts of nature in this direction. Moisture, and especially air, are both irritants, and both doubtless detrimental to the process of healing. Hence the object of eliminating the moisture by the use of hot air and excluding the external air by permanently closing the pulp-canals. I firmly believe from experience and observation that many teeth have been lost by prolonged treatment, very many times from force of habit, without regard to the classifications I have made, and that pericementitis has been produced and maintained, where this complication would not otherwise have existed.

To treat a pulp-canal without removing all the dead pulp, or to place any medicine in contact with the walls of the pulp-canal without first thoroughly evaporating the moisture with hôt air, I regard as a very great waste of time, and very bad surgery. I do not presume to deny that an abscess may be cured by using carbolic acid or creosote placed in contact with moist tubuli, but that is not proof that there is not a more excellent method by which the same results can be accomplished more surely and quickly. When you recall the difficulties with which you contend in disinfecting the tubuli when filled with moisture, as compared with the results obtainable after thoroughly evaporating the moisture from the pulp-canals and tubuli with hot air, I am sure you will discard the former practice and adopt the latter. One such effort, thoroughly made, will convince any man that the tangible results from using hot air alternated with alcohol, as compared with the use of either carbolic acid or creosote, are so much more satisfactory that I am quite willing to take my chances on his returning to the use of the two remedies last named.

Another reason for not using carbolic acid in treating these conditions is the fact that it coagulates the organic contents and does not penetrate the dentinal tubules, and certainly will have a tendency to close the foramen, preventing free drainage, which is so essential in treatment of the class of cases now being considered and where a fistulous opening does not exist. As an injection through a fistulous opening, if one is used for the purpose of stimulating granulations, I should recommend boracic acid or sulphate of zinc. At one period in my experience I assisted for five years a surgeon with an extensive practice, and the one fact which he most impressed upon me was to not indulge in what he termed "meddlesome treatment." He said he believed this to be a universal fault among surgeons, and my observation since that time convinces me that his criticism may be truthfully applied to dental surgeons.

In considering the treatment of pulpless teeth with fistulæ, one very common error into which many have fallen is the conclusion which they universally seem to have reached, that an abscess will not occur on a tooth where the pulp-canal has been perfectly filled. I indulged in this erroneous belief for many years, until my attention was called to a case one night to lance an abscess, which I did not again see for two weeks, and when seen the opening made had absolutely closed and the tooth was normally comfortable. This condition of things led me to ask myself, Was this abscess caused by an imperfect filling in the pulp-canal? If so, and the source of the abscess yet remains, why should the fistulous opening close up so thoroughly and quickly without the use of medicine? I

consulted a number of professional friends, and they all emphatically assured me that an abscess could not occur on a tooth where the pulp-canal had been thoroughly filled, but none of them could give a reason for it, nor could they for its being cured without the usual treatment. I was unable to explain the origin of the abscess in this case, but I soon reached the conclusion that it was not caused by an imperfectly-filled pulp-canal. About this time another case was presented, which excited my curiosity and led me to make an operation, which I have made many times since with great satisfaction to myself and patients. This was a case of a pulpless tooth which had been filled fifteen years and had all the surface indications of being a most perfect operation. One year previous to the time I saw it, it had abscessed and the fistula had never closed. My first thought was, Is this another case in which possibly there is a defective filling in the pulp-canal? The character of the operation made this scarcely possible, as it was most beautifully done.

The next question was, If the pulp-canal is perfectly filled, why is this fistula maintained? It then occurred to me that in both of these cases the work had been well done, but in the first mentioned there were conditions existing favorable to the process of healing, and in the last they were unfavorable, and the result was, the first had been cured without the use of medicine in two weeks, and the second had existed a year, and was, when presented to me, a case of chronic abscess. Then I said to myself, if this be the true situation as to the conditions in the pulp-canal in both of these cases, then the maintaining cause in this last one must be outside the pulp-canal, but where? Passing a probe through the fistulous opening, I detected very quickly rough margins of process, and concluding these rough margins to be the maintaining cause in this case, I proceeded to thoroughly remove with my engine and a fissure bur all evidence of rough bone, leaving a margin which could not prove a source of irritation. At this stage in my experience, I still believed in the necessity of treating an abscess even though the cause had been removed. However, I concluded to omit my former practice and note the result. To my great satisfaction, in about two weeks I found the fistulous opening closed. I saw the case at least once a year for five years subsequently, and the abscess never returned. Since these two experiences, I have strictly adhered to the following mode of practice:

If a case be presented with the pulp-canal unfilled and fistula, I thoroughly evaporate the moisture from the pulp-canal with hot air, then wash the canals with alcohol as previously described, and fill at once. If the abscess is one of recent development, I should give the case at least ten days after filling the pulp-canal, to determine whether or not the abscess was maintained by the conditions existing without the pulp-canal. If the abscess remained, I should then conclude that the maintaining cause was rough margins of bone, and should proceed as described to remove these rough margins, and have not found it necessary in such cases to resort to any further treatment. Thus, I am clearly of the opinion that two causes invariably maintain an abscess: one is the diseased condition which may exist within the nerve-canal, and the other is rough margins of process. If the abscess is of recent development, cleansing and thoroughly filling the pulp-canal as described will universally effect a cure. If the abscess has existed for several months, then removing the rough margins of process I have found to be necessary. If the maintaining cause of a recently-developed abscess was other than an unfilled pulp-canal, then simply filling the canal would not cure the abscess ; and if after the pulp-canal has been filled, it still remains, and is then cured by removing rough margins of process, then, reasoning by exclusion, I must conclude that these must have been the originating cause.

Now, if either or both of these methods, whichever the condition indicated necessary, will produce a cure, then why use carbolic acid or creosote ? I do not deny that abscesses have been cured by their use before and after filling the pulp-canal, but is such a result proof of the fact that their use is required? Extracting a valuable tooth may cure an abscess, but that is not proof that there could not have been devised a more excellent method by which the case could have been treated. There are many methods of accomplishing the same results, but to find out and adopt the best one is, I assume, our object in discussing this subject. I am an advocate of the operation of alveolotomy, where it is not possible to get access to an abscess without enlarging the apical opening, and where, if access is not gained, the patient will be obliged to suffer for many hours, and perhaps several days.

The two chief objections which have been urged against the operation of alveolotomy is, first, the injury which may be done to the surrounding tissues, and also the pain incident to the operation. As to the pain produced, I will say that by using cocaine the operation is not as much complained of by patients as the suffering which they have endured in most cases for many hours, and the prospect of relief from subsequent pain is also an incentive to have the operation performed. My personal experience is that the operation of alveolotomy produces less injury to surrounding tissues than enlarging the apical opening for the purpose of getting access to an abscess. The opportunities for seeing what you are doing in performing alveolotomy, and the fact that nature will reproduce the parts removed, as contrasted with the uncertainties which characterize the operation of enlarging the apical opening, and the additional fact that the parts destroyed by this operation have to be reproduced by mechanical means, ought not to leave any doubt as to the fact that enlarging the apical opening in any case is a surgical operation which cannot be too strongly condemned. The only time in which I have found it necessary to extract and replant a tooth in the past five years for the cure of abscess was that of a superior central incisor in which the apical opening had been enlarged for the purpose of getting access to the abscess, and had been subsequently treated for thirteen months unsuccessfully by a gentleman of skill and large experience. Hence my belief that this operation ought never to be performed.

There is also the additional fact that nature finally relieves the patient by making an opening practically the same as would result from the operation of alveolotomy, and this surely is an excellent reason for anticipating nature in such cases and giving the patient relief. I have no doubt that there are many oral surgical operations which are very imperfectly performed. Three or four cases will illustrate my reasons for this conclusion: In one all the lower teeth to the left of the symphysis were lost by an abscess being badly treated on a left inferior first molar in the mouth of a little girl twelve years of age. The patient was brought to me after being treated seven months, and I considered myself fortunate to have been able to save the base of the jaw and the teeth on the opposite side. Another was a case of fracture of the lower maxilla, which had not been properly treated for four weeks when it was placed in my charge. The third was where a blow on the left side of the face had produced necrosis and the loss of the lower molars, and a fracture about one-half inch to the right of the symphysis. This case was presented to me in consultation three months after the injury. The fractured portions had not been adjusted, and four fistulous openings were discharging through the cheek. Such cases prove most conclusively that the field of oral surgery is not as yet given the attention which it demands by the dental nor by the medical profession. In proof of this statement, I will mention the fact that the last case described had been treated for three months previous to the time the patient was brought to me, by two graduates of Jefferson Medical College, one of them being a gentleman of large experience. Right here the question naturally comes up as to the position the dental surgeon is supposed to occupy. Where does his work cease and that of the general surgeon begin? The field of oral surgery is one in which there are many opportunities for doing much good and greatly relieving humanity. Thus far, but few among the great number of dentists seem to fully appreciate its true significance and usefulness. It is a type of surgery which requires keen perceptive faculties, and the operator must possess a clear head, an honest heart, and a steady hand; and head, heart, and hand must work harmoniously together.

The advocates of carbolic acid and creosote claim that their use is necessary to destroy the pus-sac surrounding an alveolar abscess. They also claim that the destruction of this sac is essential to the cure. If the use of these remedies and the destruction of this sac is necessary, then why not necessary in the cure of abscess in any other portion of the body? I challenge any gentleman present to produce an authority on surgery in which the destruction or removal of this sac is advocated, or where it is claimed this has anything to do with maintaining an abscess on any other part. And yet in alveolar abscess, where the tissues surrounding the disease are peculiarly non-vascular, and where, if the osseous tissues were *not involved*, the conditions would be most favorable to the healing process, we are told by the advocates of the use of carbolic acid and creosote that they are absolutely essential to the cure of alveolar abscess for the reasons given. This sac is simply a barrier thrown out by nature to circumscribe the disease, and is not maintained by other than two causes, one of these being a diseased condition within the pulp-canal, and the other rough margins of bone. Remove these two maintaining causes, and the disease is cured, without the use of carbolic acid or creosote.

